# Motion Sensing by a Highly Sensitive Nanogold Strain Sensor in a Biomimetic 3D Environment

**DOI:** 10.1021/acsami.4c08105

**Published:** 2024-09-10

**Authors:** Shin-Da Wu, Horst Weller, Tobias Vossmeyer, Shan-hui Hsu

**Affiliations:** † Institute of Polymer Science and Engineering, National Taiwan University, No. 1, Sec. 4 Roosevelt Road, Taipei 106319, Taiwan; ‡ Institute of Physical Chemistry, University of Hamburg, Grindelallee 117, Hamburg 20146, Germany; § Institute of Cellular and System Medicine, National Health Research Institutes, Miaoli 350401, Taiwan; ∥ Fraunhofer Center for Applied Nanotechnology CAN, Grindelallee 117, Hamburg 20146, Germany

**Keywords:** Flexible electronics, gold nanoparticle, polyurethane, self-healing hydrogel, cardiomyocyte spheroid

## Abstract

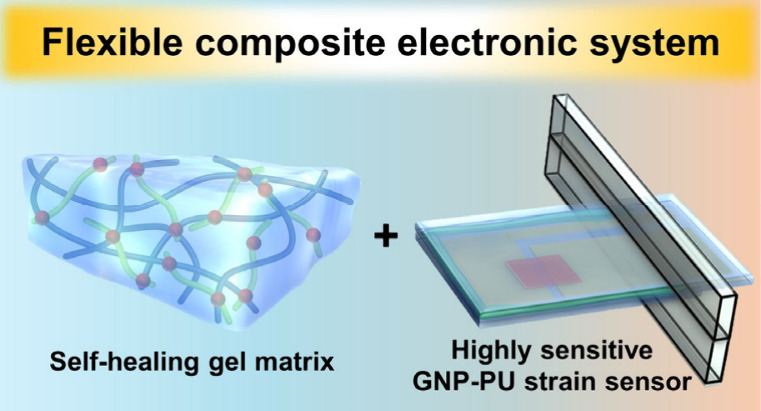

Recent advancements in flexible electronics have highlighted their potential in biomedical applications, primarily due to their human-friendly nature. This study introduces a new flexible electronic system designed for motion sensing in a biomimetic three-dimensional (3D) environment. The system features a self-healing gel matrix (chitosan-based hydrogel) that effectively mimics the dynamics of the extracellular matrix (ECM), and is integrated with a highly sensitive thin-film resistive strain sensor, which is fabricated by incorporating a cross-linked gold nanoparticle (GNP) thin film as the active conductive layer onto a biocompatible microphase-separated polyurethane (PU) substrate through a clean, rapid, and high-precision contact printing method. The GNP-PU strain sensor demonstrates high sensitivity (a gauge factor of ∼50), good stability, and waterproofing properties. The feasibility of detecting small motion was evaluated by sensing the beating of human induced pluripotent stem cell (hiPSC)-derived cardiomyocyte spheroids embedded in the gel matrix. The integration of these components exemplifies a proof-of-concept for using flexible electronics comprising self-healing hydrogel and thin-film nanogold in cardiac sensing and offers promising insights into the development of next-generation biomimetic flexible electronic devices.

## 1 Introduction

Flexible electronics have received significant interest recently due to their compatibility with human physiology, making them suitable for wearable and biomedical electronics.^1^ For instance, cantilever-based gold-polydimethylsiloxane (PDMS) strain gauges have been utilized for microphysiological measurements.^2^ In another example, strain sensors composed of cross-linked gold nanoparticle (GNP) thin films as the active conductive layer and biodegradable, waterborne polyurethane (PU) films as the flexible substrate were applied as eco-friendly wearable electronics for monitoring human pulse waveforms.^3^ However, the sensitivity of these sensors, with gauge factors of approximately 0.6 and 13, respectively, is not entirely satisfactory for detecting small deformations.^2^ To accurately measure motion in biomimetic tissues like cardiomyocyte contractility, existing methods often involve optical techniques such as video analysis, laser sensing, atomic force microscopy (AFM), and traction force microscopy (TFM).^4−6^ These methods have challenges, such as complex and time-consuming setup procedures for optical methods, potential alteration of cardiomyocyte beating patterns by AFM, and the high cost and complexity of AFM and TFM instruments. To address these limitations, electrical signal-based contractility sensors have emerged as a promising alternative.^7^ These sensors, such as strain and impedance sensors, enable facile, real-time, and long-term recording of cardiac contractility.^8^ Despite these advancements, detecting the nuanced movements in three-dimensional (3D) cardiac tissues with strain sensors remains challenging.^4^ Therefore, developing high-sensitivity flexible electronics is crucial for broader biomedical applications.

Self-healing hydrogels have received considerable attention for integration with flexible electronics due to their excellent biocompatibility and mechanical properties that mimic natural tissues.^9^ In particular, intrinsic and autonomous self-healing hydrogels are capable of repairing damages and restoring their original structures and functions without the need for external stimuli or healing agents.^10^ These hydrogels serve as an ideal medium for replicating the extracellular matrix (ECM) in biomimetic environments, providing support for cell growth and function while adapting to dynamic cellular behaviors.^4^ For instance, Song et al. demonstrated that cardiomyocytes cultured in self-healing hydrogels exhibited more oriented and elongated sarcomeres compared to those grown in permanently cross-linked hydrogels.^11^ This finding highlights the advantage of the dynamic network of self-healing hydrogel, which can stably endure continuous mechanical deformations by cardiomyocytes. Therefore, using suitable self-healing hydrogels to support the development and maturation of 3D tissues is crucial for improving the performance and applicability of biomimetic flexible electronics.^9,12^

The substrate in flexible electronics has two crucial roles: supporting the ECM (e.g., self-healing hydrogels) that nurtures biomimetic tissues and serving as a structural base for the active conductive layer.^3,13^ Therefore, the substrate must have surface properties compatible with the ECM for effective integration, which is essential for reliable sensing. Additionally, the substrate needs to be biocompatible and waterproof to maintain cell viability and sensing functionality in the cell culture medium at physiological temperature.^6^ Biomimetic 3D tissues offer a more detailed replication of structural and cellular complexities compared to two-dimensional (2D) laminar tissues, enhancing cell–cell and cell-ECM interactions and more accurately mimicking natural human tissues.^4,14−16^ These advancements in tissue engineering significantly improve the fidelity and applicability of biomimetic models in medical research and therapy.^17^

In this study, we introduce a new flexible electronic system that integrates the self-healing hydrogel matrix with the highly sensitive cantilever-based GNP-PU strain sensor, designed to demonstrate a proof-of-concept application for sensing small movements such as cardiac beating. In the system, the self-healing hydrogel functions as the cell growth matrix, providing a biomimetic 3D environment for the cells, and can relay the motion behavior of the cells to the sensing component (i.e., GNP-PU strain sensor) for measurement. The self-healing hydrogel matrix is composed of phenol-functionalized chitosan (CS-Ph) as the main chain and difunctional poly(ethylene glycol) (DF-PEG) as the cross-linker, while the GNP-PU strain sensor consists of a GNP thin film as the active conductive layer and a biocompatible, waterproof PU film as the flexible substrate. A facile, clean, and high-precision contact printing method was used for transferring the GNP film from the glass onto the PU substrate, without using cytotoxic organic solvents or chemical etchants. The fabricated GNP-PU strain sensors demonstrated high sensitivity with a gauge factor of ∼50. To assess the feasibility of the integrated platform, the hydrogel matrix was laden with 3D human induced pluripotent stem cell (hiPSC)-derived cardiomyocyte spheroids. The developed flexible electronic system was capable of detecting the contractile behavior of the cardiomyocyte spheroids, emphasizing its utility in sensing the motion within biomimetic 3D environments.

## 2 Results

**Figure 1:**
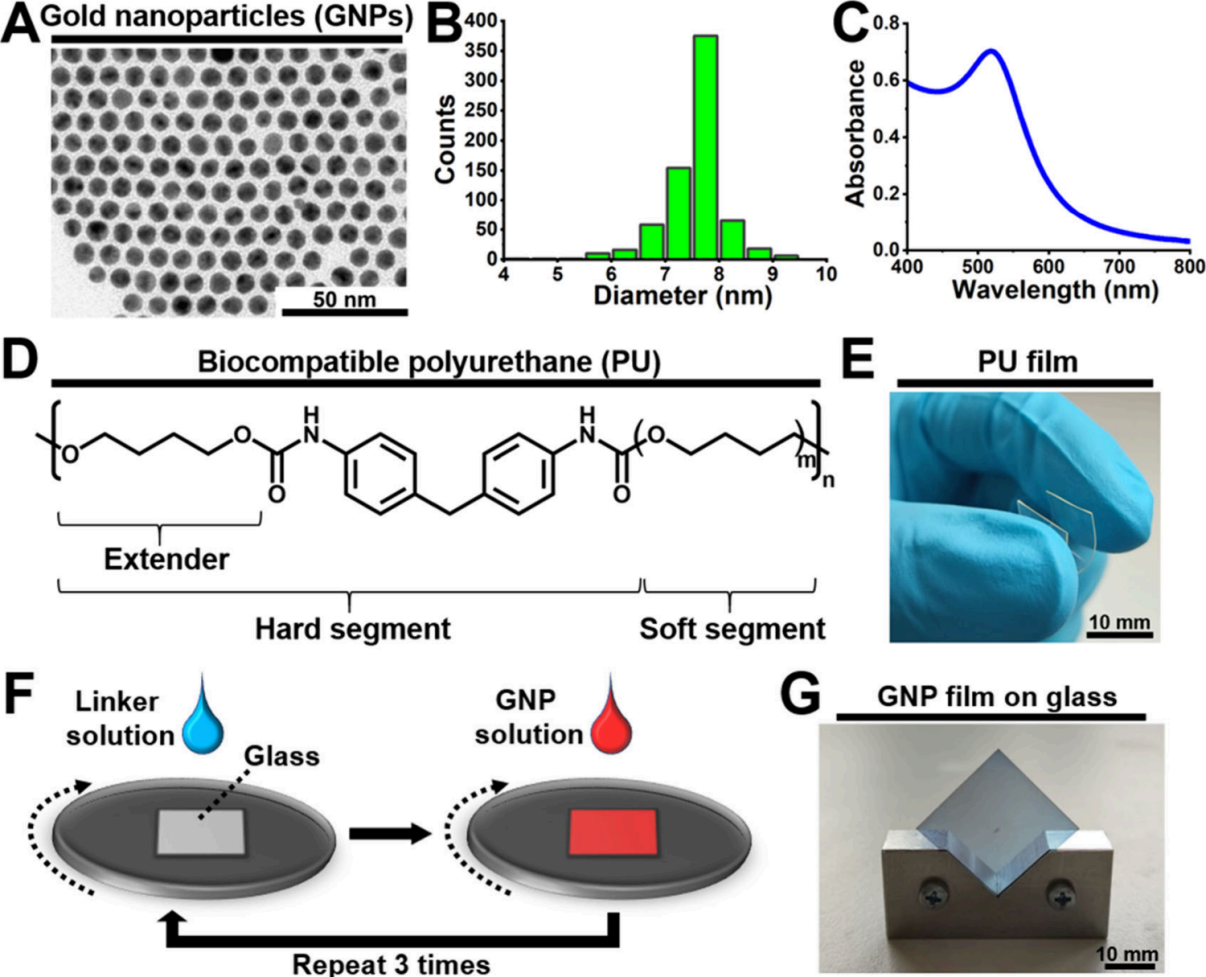
Characteristics of the two components [i.e., gold nanoparticles (GNPs) and biocompatible polyurethane (PU)] of the strain sensor and preparation of the cross-linked GNP film. (A) The transmission electron microscopy (TEM) image, (B) size distribution histogram, and (C) ultraviolet–visible (UV–vis) absorption spectrum of GNPs. (D) Chemical structure of the biocompatible PU. (E) Photograph showing flexibility of the biocompatible PU film. (F) Layer-by-layer spin-coating deposition of the 1,9-nonanedithiol (9DT) cross-linked GNP film. (G) Optical appearance of the fabricated GNP film deposited onto the glass substrate.

Figure 1 details the characterization of two key elements of the strain sensor: GNPs and biocompatible PU, along with the preparation of the cross-linked GNP film. A transmission electron microscopy (TEM) image of the synthesized GNPs is shown in Figure 1A. The TEM images indicated that the GNPs were spherical, with an average diameter of 7.5 ± 0.6 nm (Figure 1B). Additionally, the ultraviolet–visible (UV–vis) absorption spectrum of the GNPs showed the localized surface plasmon resonance peak at the wavelength of ∼520 nm (Figure 1C). Figure 1D illustrates the molecular structure of the biocompatible PU. The PU film, produced by casting, had a thickness of ∼230 μm and was featured high flexibility, as displayed in Figure 1E. The fabrication of the GNP film on a glass slide was carried out using a layer-by-layer spin-coating process, as shown in Figure 1F. During the process, 1,9-nonanedithiol (9DT) cross-linker solution and GNP solution were alternately applied onto the spinning glass slide. This spin-coating procedure was repeated for three cycles to complete the fabrication of the cross-linked GNP film. A photograph of the resulting cross-linked GNP film on the glass substrate is shown in Figure 1G. The GNP film exhibited a bluish color.

**Figure 2:**
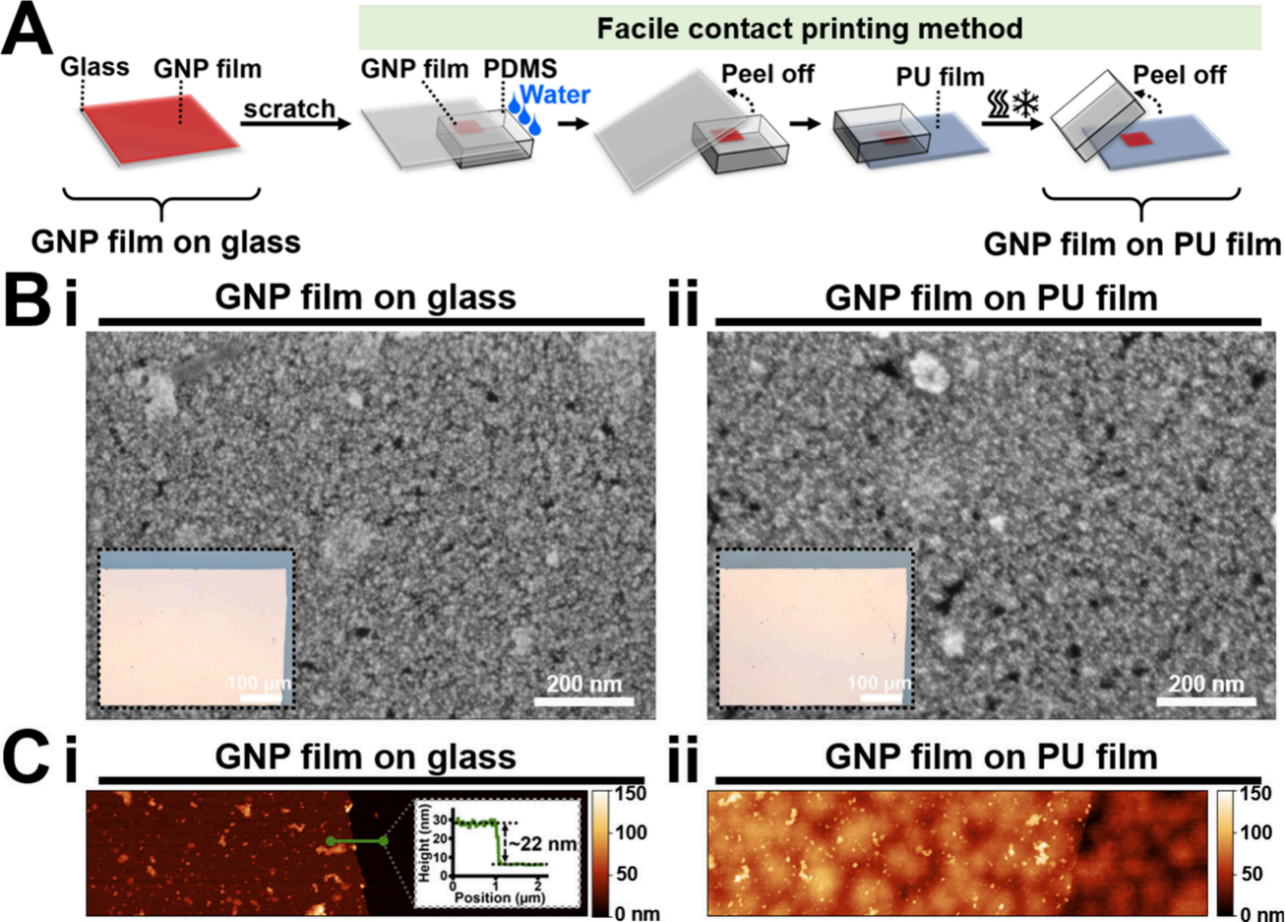
Printing procedures for transferring the GNP film onto the biocompatible PU film and the surface morphology of the GNP film before (i.e., on the glass substrate) and after (i.e., on the PU film) the transfer. (A) Schematic illustration showing the facile contact printing method employed for transferring the GNP film onto the PU film. (B) Optical microscopy images (insets) and scanning electron microscopy (SEM) images of the GNP film on the (i) glass substrate and (ii) PU film. (C) Two-dimensional (2D) atomic force microscopy (AFM) images of the GNP film on the (i) glass substrate and (ii) PU film. The inset in (Ci) shows the height profile of the GNP film.

For transferring the cross-linked GNP film from the glass substrate to the biocompatible PU film, a facile and clean contact printing method was employed, illustrated in Figure 2A. First, the GNP film on the glass substrate was scratched using a sharp blade to create a square shape with a size of 4 mm^2^. Then, a PDMS stamp was applied to the surface of the GNP film to aid the transfer. A small volume of water (∼3 μL) was dripped to the interface between the glass and the GNP film. The GNP film was then transferred onto the PDMS stamp by gently peeling off the glass substrate. Subsequently, the PDMS stamp, now bearing the GNP film, was placed onto the biocompatible PU film. Following a heating and cooling cycle, the PDMS stamp was easily removed, allowing for the successful transfer of the GNP film onto the PU film. Figure 2B, C and Figure S1 demonstrate the surface morphology of the GNP film before (i.e., on the glass substrate), during (i.e., on the PDMS stamp), and after contact printing (i.e., on the PU film). The optical images in the inset of Figure 2B show that the geometry of the film remained intact after contact printing, with no visible cracks when transferred onto the PU film. The scanning electron microscopy (SEM) images in Figure 2B display the granular structure made of cross-linked GNPs, confirming consistent surface morphology across both substrates. The 2D AFM images in Figure 2C demonstrate the thickness and surface roughness of the GNP film. The thickness of the GNP film was ∼22 nm, as shown in the inset of Figure 2Ci. The PU film exhibited greater roughness than the glass (Figure S2 and Figure 2C), resulting in higher average roughness for the GNP film on the PU film (∼11 nm) compared to that on glass (∼5 nm). Despite this finding, the overall morphology of the GNP film remained unchanged postprinting.

**Figure 3:**
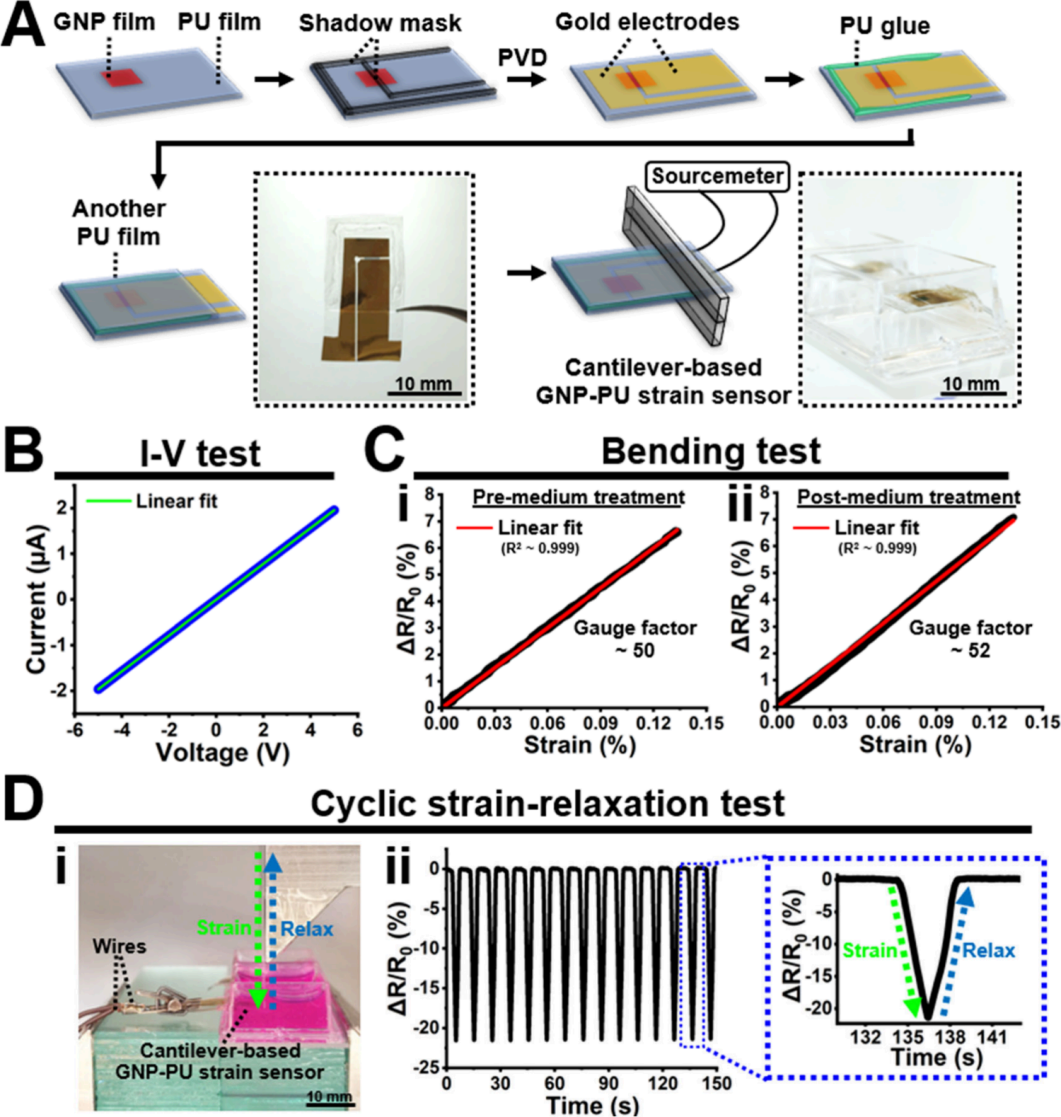
Fabrication and characterization of the cantilever-based GNP-PU strain sensor. (A) Schematic illustration showing the fabrication procedure of the cantilever-based GNP-PU strain sensor. A linear fit to the data is shown as the green solid line. (B) The current–voltage (*I*–*V*) curve of the sensor. (C) Results of bending tests: relationship between relative resistance change (Δ*R*/*R*_0_) and strain of the sensor (i) before and (ii) after the cell culture medium treatment for 2 days at 37 °C. Linear fits to the data are shown as red solid lines. (D) (i) Setup to conduct 15 strain-relaxation cycles and (ii) results of the cyclic strain-relaxation test on the cantilever-based GNP-PU strain sensor.

Figure 3A outlines the process used to fabricate the cantilever-based GNP-PU strain sensor. Initially, gold electrodes were applied onto the GNP-PU film through physical vapor deposition (PVD), utilizing a shadow mask. The deposited gold electrodes had a thickness of ∼100 nm, with a gap of ∼230 μm between them. Subsequently, a PU glue with a concentration of ∼35 wt % was employed to bond an additional PU film to the GNP-PU strain sensor. The final step involved fixing the GNP-PU strain sensor in a tissue culture chamber, resulting in the formation of the cantilever-based GNP-PU strain sensor. An image of the completed cantilever-based GNP-PU strain sensor is shown in the figure inset.

Figure 3B-D presents the characterization of the GNP-PU strain sensor. The current–voltage (I–V) test of the sensor demonstrated ohmic conductivity (Figure 3B), with a conductance of ∼0.39 μS. The resistive responses of the sensor to strain were evaluated through bending and cyclic strain-relaxation tests. In the bending tests, the relative resistive response (Δ*R*/*R*_0_) of the sensor exhibited a nearly linear change with strain. The gauge factor of the sensor, regarded as its sensitivity, is calculated as the slope of the Δ*R*/*R*_0_ versus strain graph. Results showed that the gauge factor before and after the cell culture medium treatment was ∼50 and ∼52, respectively (Figure 3C). During the cyclic strain-relaxation test (Figure 3D), the sensor was subjected to 15 strain-relaxation cycles and displayed good repeatability of the sensor’s response characteristics.

**Figure 4:**
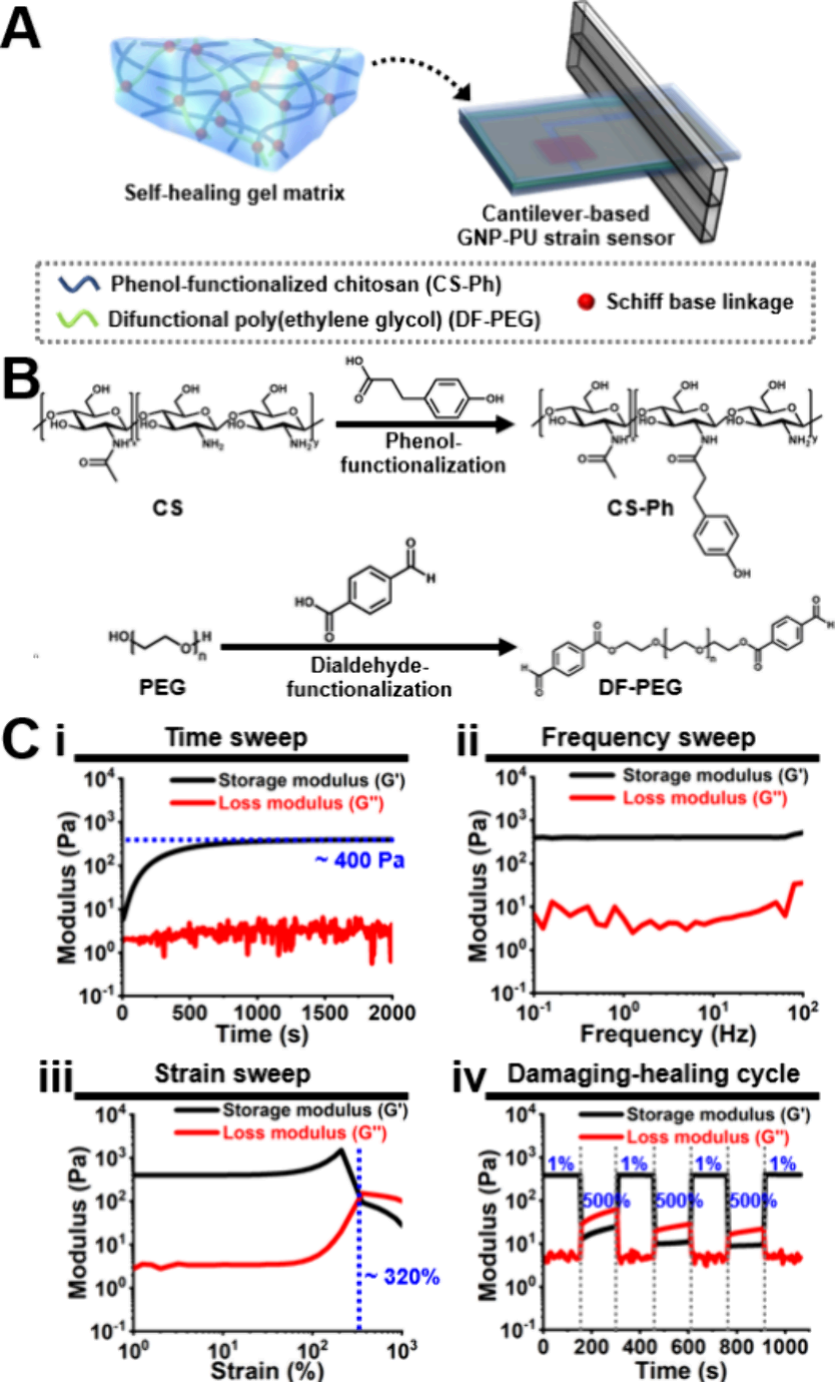
Characterization of the hydrogel matrix within the flexible electronic system. (A) The flexible electronic system is composed of the self-healing hydrogel matrix and the cantilever-based GNP-PU strain sensor. The gel matrix forms through the reaction of phenol-functionalized chitosan (CS-Ph) and difunctional poly(ethylene glycol) (DF-PEG) via dynamic Schiff base linkage. (B) Synthesis routes and chemical structures of CS-Ph and DF-PEG. (C) Rheological properties of the hydrogel matrix. (i) Time-dependent moduli after sample loading, measured at 1% strain and 1 Hz. (ii) Frequency-dependent moduli after gel stabilization, measured at 1% strain. (iii) Strain-dependent moduli after gel stabilization, measured at 1 Hz. (iv) Damaging-healing cycles measured at 1 Hz through the continuous step strain changes.

A flexible electronic system, integrating a gel matrix with a highly sensitive cantilever-based GNP-PU strain sensor, was specifically designed to demonstrate a proof-of-concept application of sensing cardiac beating, as illustrated in Figure 4A. The gel matrix is a self-healing hydrogel that can conveniently be loaded with cells. The chemical structures of the two components (i.e., CS-Ph and DF-PEG) within the self-healing hydrogel are presented in Figure 4B. CS-Ph is synthesized by the conjugation of phloretic acid to chitosan through carbodiimide chemistry, while DF-PEG is synthesized via Steglich esterification. The self-healing hydrogel is then fabricated using CS-Ph as the main chain and DF-PEG as the cross-linker, forming dynamic Schiff base linkages.

The rheological properties of the fabricated self-healing chitosan-based hydrogel are shown in Figure 4C. In the time sweep, the gel point [crossover point of the storage (*G*′) modulus and the loss (*G*′′) modulus] occurred before the rheological analysis, indicating the fast gelling property of the self-healing hydrogel (Figure 4**Ci**). The *G*′ value of the hydrogel underwent a significant increase in the initial gelling stage. After ∼500 s, this value became stabilized, eventually reaching a steady state of ∼400 Pa. Meanwhile, the *G*′′ value of the hydrogel oscillated with time. In the frequency sweep, the hydrogel exhibited low frequency dependence of *G*′ and *G*′′, indicating the inherent solid-like relaxation behavior (Figure 4Cii). In the strain sweep, the critical strain point for transitioning from the gel state to sol state (i.e., structural damage) was ∼320% (Figure 4Ciii). The self-healing property of the hydrogel was assessed by applying strains alternately, switching between a high strain that exceeded the critical strain point and a lower strain of 1% (Figure 4Civ). Below 1% strain, *G*′ was higher than *G*′′ and both moduli remained constant over time. At the higher strains of 500%, a *G*′-*G*′′ crossover had occurred, indicating a sol state with *G*′′ greater than *G*′. Upon strain reversal, the values of *G*′ and *G*′′ quickly returned to their initial levels. This repeatable rheological behavior after several damaging-healing cycles confirmed the self-healing nature of the chitosan-based hydrogel.

**Figure 5:**
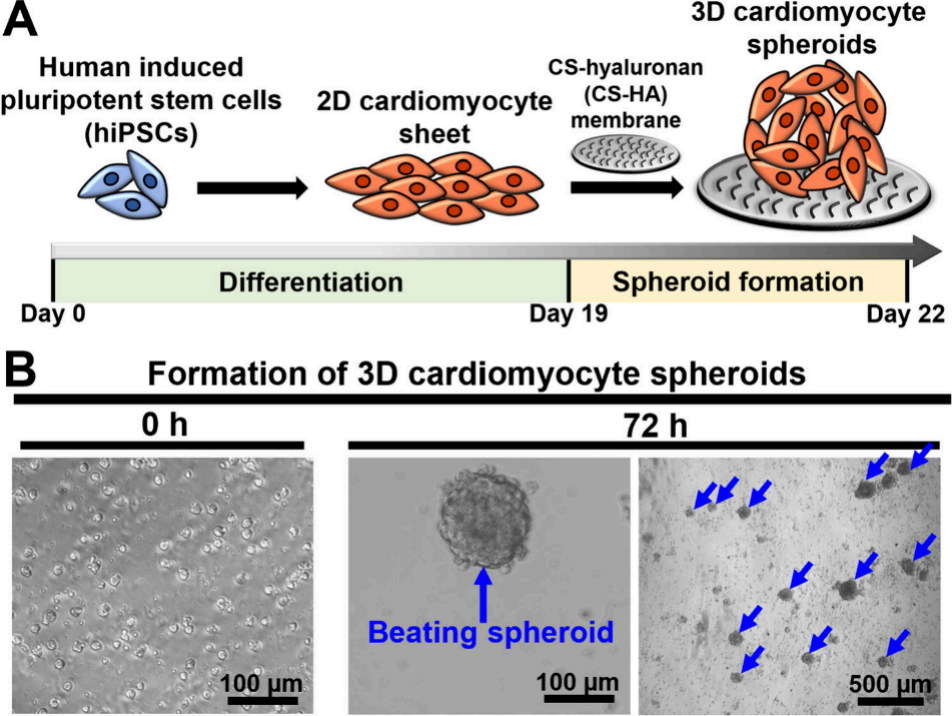
Preparation of 3D cardiomyocyte spheroids. (A) Schematic illustration of the timeline of differentiation from human induced pluripotent stem cells (hiPSCs) to a 2D cardiomyocyte sheet. Subsequently, the 2D cardiomyocyte sheet was dissociated and self-assembled into 3D cardiomyocyte spheroids on the CS-hyaluronan (CS-HA) plate. (B) Optical microscopy images of the formation of 3D cardiomyocyte spheroids on the CS-HA plate at 0 and 72 h.

To mimic the 3D cardiomyocyte beating, the fabrication process of the 3D cardiomyocyte spheroids is illustrated in Figure 5A. Initially, hiPSCs were grown according to the procedure outlined in the commercially available cardiomyocyte differentiation kit, and the hiPSCs underwent differentiation to form a 2D cardiomyocyte sheet. Subsequently, the cardiomyocyte sheet was dissociated into single-cell cardiomyocytes and seeded onto the chitosan-hyaluronan (CS-HA) membrane (0 h), as shown in Figure 5B. After further culture for 72 h, the individual cardiomyocytes spontaneously assembled into the 3D and beating cardiomyocyte spheroids. The 3D spheroids formed had diameters ranging approximately between 90 to 150 μm.

**Figure 6:**
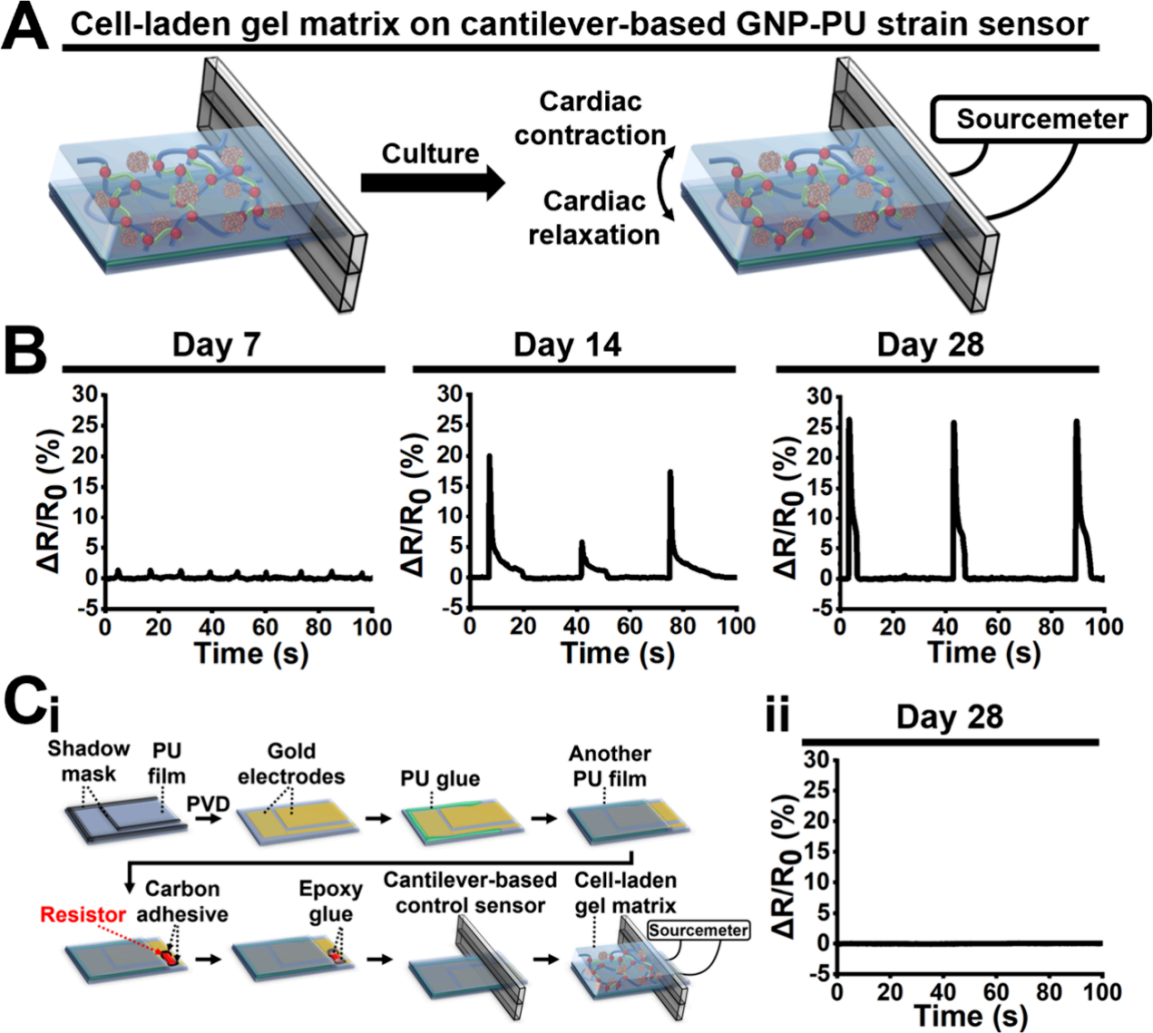
Proof-of-concept application of the flexible electronic system. (A) Schematic illustration showing the application of the cantilever-based GNP-PU strain sensor to measure the contraction/relaxation of cardiomyocyte spheroids within the gel matrix. (B) Δ*R*/*R*_0_ signals of the cantilever-based GNP-PU strain sensor at 7, 14, and 28 days. (C) (i) Fabrication procedure of the cantilever-based control sensor. The design concept of the control sensor is to substitute the GNP film with a nonstrain-responsive resistor, with the aim to confirm that the results in (B) are attributed to the strain behavior of cardiomyocyte spheroids. (ii) Δ*R*/*R*_0_ signal of the cantilever-based control sensor at day 28.

The proof-of-concept application of the flexible electronic system in a biomimetic 3D environment is shown in Figure 6A, where the GNP-PU strain sensor effectively captured the contraction and relaxation cycles of 3D hiPSC-derived cardiomyocyte spheroids embedded in the self-healing hydrogel. Cardiac contractile behavior was precisely monitored using the cantilever-based GNP-PU strain sensor. Figure 6B shows the real-time Δ*R*/*R*_0_ signal traces of contraction and relaxation characteristics of cardiomyocyte spheroids measured at days 7, 14, and 28. Initially, the cultured cardiomyocyte spheroids showed a measurable beating rate at an early stage of the culture period (day 7). As the culture period progressed, the beating rate of the cardiomyocyte spheroids gradually decreased, yet in parallel, their contraction force increased. By day 28, the contraction force stabilized at its highest level. To ensure that the Δ*R*/*R*_0_ signals in Figure 6B originated from strain-induced responses rather than electrophysiological signals from cardiac contractions, a cantilever-based control sensor has been specifically designed and fabricated (Figure 6Ci). This control sensor was modified from the standard design by replacing the GNP film with a nonstrain-responsive resistor. The experiment demonstrated that for cardiomyocyte spheroids cultured on the cantilever-based control sensor over 28 days, no Δ*R*/*R*_0_ signal was detected by the sourcemeter (Figure 6Cii). This result confirms that the Δ*R*/*R*_0_ signals in Figure 6B indeed resulted from strain changes caused by the contractile behavior of cardiomyocytes.

## 3 Discussion

Thin-film strain sensors have gained much attention for cardiac sensing due to the flexibility.^7^ These sensors typically consist of two components: an active conductive layer and a flexible substrate.^18^ The active conductive layer, made from organic and/or inorganic nanomaterials, provides the functional sensing properties.^19^ Among various metal nanoparticles, GNPs are considered the most appropriate for in vitro and in vivo biomedical applications due to their biocompatibility.^20^ GNP thin films, known for the stimuli-responsive charge transport properties, are commonly used as the active conductive layer in high-performance strain sensors.^3,13^

PDMS is one of the most popular flexible substrates for cardiac sensing due to its nontoxicity, chemical inertness, low Young’s modulus, and optical transparency.^1,6^ For instance, Wang et al. used PDMS in a muscular thin-film platform to measure contractility in cardiomyocyte monolayers, and Kim et al. used a PDMS cantilever to measure drug-induced changes in a 2D cardiomyocyte model.^21,22^ However, PDMS presents some limitations, which particularly the hydrophobicity can hamper the effective bonding between the surface of PDMS and a hydrophilic hydrogel.^2,23^ Therefore, adherence of cardiomyocyte spheroid-laden hydrogel to a PDMS substrate is not feasible. Furthermore, the inert nature of PDMS leads to poor affinity toward metal nanoparticle surfaces, which is detrimental for the robust attachment of the active conductive GNP layer.^24,25^ An additional drawback of PDMS is its high absorption rate of small molecules, a factor that is particularly significant in biomedical applications.^6^ For instance, Zhao et al. and Toepke et al. reported that PDMS absorbs hydrophobic drugs, thereby impacting short-term and long-term drug screening studies.^26,27^ In contrast, Pellethane 2363 80A, a thermoplastic type of PU, offers distinct advantages over PDMS, including superior resistance to water and chemicals, biocompatibility [Food and Drug Administration (FDA) approved], and processability.^16,28,29^ The flexible substrate fabricated from Pellethane 2363 80A was hydrophilic (contact angle ∼60°).^30^ Therefore, in this study, the PU substrate demonstrated good adherence with the cell-laden chitosan-based hydrogel, facilitating reliable sensing of cardiac contractility. Furthermore, the Young’s modulus of the PU film was ∼4.1 MPa (based on the commercial data sheet), which is considered low enough for sensing applications.^3^ Moreover, the nitrogen atoms present in the PU promote enhanced interactions with metallic nanomaterials.^31^ For instance, Kim et al. demonstrated that the interaction between GNPs and PU results in the formation of highly stretchable nanoparticle conductors.^32^ In our previously published study, the eco-friendly GNP-PU strain sensors showed high stretchability and good durability.^3^

The fabrication of flexible thin-film strain sensors involves integrating the active conductive layer with the flexible substrate, a critical step for sensor functionality and reliability.^3,7^ Various printing methods have been developed for this purpose, categorized into contact and noncontact types.^33^ Noncontact methods, like inkjet and electrohydrodynamic printing, face challenges such as nozzle clogging, low printing speeds, limited ink viscosity range, and the use of cytotoxic organic solvents, which are unsuitable for biomedical applications.^34,35^ Contact printing methods, like the peel-and-stick process and poly(methyl methacrylate) (PMMA)-mediated transfer printing, also involve the use of organic solvents.^36,37^ Other methods, such as the wedging transfer process, can cause cracks or wrinkles during transfer, degrading sensor performance.^19^ The lift-off by etching method uses chemical etchants that can damage the active layer or alter the properties of the flexible substrate.^38^ To overcome these challenges, we employed a solvent-free and facile contact printing method, previously developed by our group,^3^ to fabricate the GNP-PU strain sensors (Figure 2A).

The gauge factor of a strain sensor is crucial for evaluating its sensitivity by measuring resistance changes due to mechanical strain. For example, Lind et al. developed carbon black-thermoplastic PU strain sensors designed for cardiac microphysiological devices, achieving a gauge factor of 2.56.^16^ For instance, Dong et al. and Kim et al. from the same team utilized metal strain sensors to monitor cardiac contractility and assess the effects of drugs, but the gauge factor was low (<5).^15,22^ It is critical to develop highly sensitive sensors to enhance the measurement accuracy.^39^ To meet this need, Kim et al., also from the same team, fabricated a microcrack-based metal-PDMS strain sensor that achieved a high gauge factor of ∼100 through initially applying a predefined strain of 2%.^40^ In the present study, the GNP-PU strain sensor exhibited a higher gauge factor (∼50; Figure 3C) compared to previously published eco-friendly GNP-PU strain sensors (with a gauge factor of ∼13) comprising biodegradable, waterborne PU,^3^ thereby indicating a higher sensitivity in response to strain. Unlike typical crack-based sensors that require preapplied strain to achieve high sensitivity,^39,40^ our sensor achieves comparable performance without the necessity to induce microcracks. We attribute the notably higher sensitivity of the current GNP-PU strain sensor to the rippled surface topology of the PU film (Figure 2Cii and Figure S2). This unique rippled structure resulted from the microphase separation between the soft and hard segments of Pellethane.^41^ During stretching, localized stress concentration occurred in the GNP film on the PU substrate, potentially forming nanoscale cracks at junctions, which contribute to the high sensitivity of the GNP-PU strain sensor.^39,42,43^ The sensor demonstrated positive resistive changes due to the increased interparticle spacing of the GNP film when bent in a four-point bending setup (Figure 3C).

To further demonstrate a proof-of-concept application, we designed the highly sensitive GNP-PU strain sensor in a cantilever format to efficiently monitor cardiac contractility (Figure 3A).^7^ The contraction of cardiomyocytes generates surface stress that causes mechanical deformation on the flexible cantilevers,^4^ which can be monitored by the embedded strain sensors. In the cantilever-based GNP-PU strain sensor, the GNP film was positioned on one side of the upper PU film, while the opposite side of the PU film made contact with cardiomyocytes. Therefore, during the cyclic strain-relaxation test (Figure 3D), the sensor exhibited negative resistive responses due to the reduced interparticle spacing of the GNP film when strain was applied by the bending setup. Furthermore, the cantilever-based GNP-PU strain sensors maintained sensing functionality in the cell culture medium, indicating the waterproofing property suitable for cellular sensing applications.

Hydrogels with tunable elastic moduli have been reported in combination with flexible cantilevers, serving as ECM and enhancing the contraction-induced cantilever deflection.^4,44^ Chitosan, a natural biomaterial, is often used in cardiac regeneration for its biocompatibility, biodegradability, and antimicrobial properties.^45,46^ Through chemical modification, chitosan can be tailored to enable customized properties. For instance, chitosan functionalization with phenol enhances its water solubility at neutral pH and accelerates the cross-linking rates, aiding in avoiding the use of acidic solution and ensuring more uniform cell distribution within the CS-Ph hydrogel.^47,48^ Further, integrating chitosan with other biomaterials can improve the functionality, such as the self-healing property, which enhances the resilience and structural integrity of the hydrogel.^49^ PEG hydrogels are well-known for the biocompatibility and capability of chemical modification. Specifically for cardiac applications, PEG hydrogels embedded with cardiomyocytes were shown to preserve sarcomeric integrity and t-tubular structure.^50^ In the present study, we prepared a self-healing chitosan-based hydrogel composed of CS-Ph and DF-PEG (Figure 4B, C), which was previously developed by our group for 3D bioprinting.^47^ Due to the fast gelling of this hydrogel and injectability after gelation,^47^ we used it as a dynamic hydrogel for supporting hiPSC-derived cardiomyocyte spheroids. The self-healing property was associated with the breaking and reforming of reversible dynamic Schiff base linkages in the hydrogel without external triggers.^47,48^

Among the various methods for generating 3D spheroids, using CS-HA membranes on culture plates is particularly attractive due to its simplicity and cost-effectiveness.^51^ HA, a major ECM component in the human body, is a natural anionic polymer widely used in biomedical applications for its biocompatibility. Previous studies have shown that the carboxyl groups of HA can graft with the amine groups of chitosan, promoting the formation of 3D spheroids from 2D stem cells.^52^ The developed 3D biomimetic spheroids can be applied in various biomedical fields in vitro such as regenerative medicine, disease modeling, and drug screening.^53^ In the present study, we have successfully utilized CS-HA membranes to facilitate the self-assembly of 2D cardiomyocytes into 3D beating cardiomyocyte spheroids (Figure 5). The use of 3D cardiomyocyte spheroids derived from hiPSCs provides a scalable and human-relevant cell source.^54^ Further, hiPSC-derived cardiomyocyte spheroids from patients with specific genotypes can closely mimic human disease phenotypes in vitro, offering precise insights into personalized cardiac physiology.^55^

We have further demonstrated a proof-of-concept application of the flexible electronic system for evaluating the contractile behavior of cardiomyocyte spheroids by integrating the spheroid-laden self-healing chitosan-based hydrogel with the cantilever-based GNP-PU strain sensor. The results showed that the sensor exhibited positive resistive responses due to the contractile behavior of cardiomyocyte spheroids, which caused an increase in the interparticle spacing of the GNP film. As the culture period increased, cardiomyocyte spheroids showed increased maturation, reflected by a decreased beating rate and increased contractility (Figure 6B).^6^ The contractile stress exerted by the cardiomyocyte spheroids on the sensor at day 28 was ∼20 kPa, calculated using the gauge factor formula (ΔR/R_o_ = gε) and Hooke’s law (σ = Eε), where g is the gauge factor of the strain sensor, ε represents strain, σ indicates stress, and E is the Young’s modulus of the PU film. The contractile stress estimated in our study falls within the range reported for similar systems,^56−58^ as shown in Table S1. In comparison to the contractile stress in heart muscles, which ranges from 40 to 80 kPa,^59^ the relevant studies on 3D cardiac tissues show lower values (2–28 kPa).^56−58^ Further, it should be noted that the actual contractile stress of the cardiomyocyte spheroids in our study may be lower than the calculated value, as the Young’s modulus of the flexible substrate may decrease due to slight swelling in the 37 °C cell culture medium. The cardiac behavior trends and signals observed align closely with those reported in published heart-on-a-chip literature,^16,40^ suggesting that our flexible electronic system holds significant potential for future development as a heart-on-a-chip platform for drug screening applications.

Cellular morphology changes at different time points were analyzed to compare cardiomyocyte spheroids cultured in the 3D gel matrix with those in the 2D environment (i.e., on the Petri dish) (Figure S3). The results showed that as the culture period increased, the cardiomyocyte spheroid within the self-healing hydrogel became more compacted, implying greater maturity and increased contractile force,^60^ which aligns with the trends observed in spheroid beating as measured by the GNP-PU strain sensor (Figure 6B). The size of the cardiomyocyte spheroid remained similar due to the ongoing fusion of neighboring cardiomyocytes into the spheroid. In contrast, the spheroid cultured on the Petri dish significantly decreased in size (from ∼153 μm to ∼108 μm) and exhibited a loosened morphology at day 28, indicating an unfavorable environment for the development of the cardiomyocyte spheroid. Moreover, the viability of the cardiomyocyte spheroids, assessed by live/dead cell staining, revealed that all spheroids survived with only a few dead cells present at the periphery after 28 days of culture (Figure S4). In addition, cardiomyocyte spheroids immunostained for cardiac troponin T were observed using confocal laser scanning microscopy to reveal their 3D distribution within the gel (Figure S5). The resulting 3D confocal images confirmed that the cardiomyocyte spheroids were distributed inside the gel after 28 days of culture.

We attribute the ability of our flexible electronic system to detect cardiac beating to two key factors: the biomimetic self-healing hydrogel and the highly sensitive cantilever-based GNP-PU strain sensor. The selection of an appropriate biomimetic self-healing hydrogel is crucial, as it must efficiently transmit the contraction forces from cardiomyocyte spheroids to the strain sensor without dissipation, ensuring accurate detection.^9^ This capability may arise from the soft characteristics (*G*′ ∼ 400 Pa) of the self-healing chitosan-based hydrogel and its deformation properties, with the force-induced strain occurring within the elastic region of the gel (Figure 4C). Additionally, Song et al. demonstrated that the uniformly distributed network of the self-healing hydrogel is vital, ensuring the continuous transmission paths that are crucial for the maturation of cardiomyocytes.^11^ In the present study, the fast gelling property of the self-healing chitosan-based hydrogel ensures a uniform and transmissible matrix.^47,61^ Furthermore, the dynamic Schiff base linkages classify the chitosan-based hydrogel as an adaptable type that can be locally modified to accommodate intricate cellular functions (e.g., cell–cell interactions), in contrast to permanently cross-linked hydrogels.^62−64^ Taken together, the adaptable chitosan-based hydrogel with its soft characteristics and uniformly distributed network may enable individual cardiomyocyte spheroids to interact and move uniformly, promoting maturation. Moreover, the use of a highly sensitive strain sensor is critical to detect the subtle forces transmitted through the self-healing hydrogel.^4^ Future work will focus on optimizing the self-healing hydrogel by exploring varying strengths and self-healing rates to advance self-healing hydrogel bioelectronics.^9^

In addition to cardiomyocytes, the human heart contains essential supporting cells, including cardiac fibroblasts, endothelial cells, smooth muscle cells, and immune cells.^65^ These supporting cells are vital for maintaining heart homeostasis, facilitating repair, producing ECM, and ensuring nutrient delivery.^65^ Therefore, coculturing cardiomyocytes with supporting cells in 3D in vitro cardiac models is important for a more comprehensive understanding of the complex cellular interactions.^4,6^ The CS-HA membrane for generating 3D spheroids can be conveniently extended to a coculture system and create spheroids with multiple cell types,^51^ which is promising for future coculture of cardiomyocytes and supporting cells. In addition, multimaterial 3D printing technology enables the facile fabrication of customized flexible electronic system.^16^ The PU substrate, GNP film, and the self-healing chitosan-based hydrogel all have potential for 3D printability, as previously reported.^16,47,66^ Furthermore, the 3D bioprintability and photoresponsiveness of the chitosan-based hydrogel may allow the creation of unidirectional microfilament networks using filament light biofabrication,^44,47^ which can guide cells to form aligned tissues.

## 4 Conclusion

This study has successfully demonstrated the development and application of a highly sensitive nanogold strain sensor integrated within a biomimetic 3D environment for motion sensing, particularly focusing on cardiac contractility. The sensor, featuring a thin film of cross-linked GNPs on a PU substrate, exhibited remarkable sensitivity and stability, with a gauge factor of ∼50. This high sensitivity is crucial for accurately detecting the nuanced movements of hiPSC-derived cardiomyocyte spheroids embedded within a self-healing chitosan-based hydrogel. The hydrogel effectively mimics the dynamics of the ECM, supporting the growth and maturation of the cardiomyocyte spheroids while enabling the dynamic monitoring of their contractile behavior. Our findings highlight the potential of integrating flexible electronics with biomimetic 3D models to enhance the fidelity and applicability of biomedical devices. In particular, choosing PU as the flexible substrate for the nanogold strain sensor offers four major advantages: (1) It exhibits good affinity with GNPs, ensuring durability; (2) The GNP-PU strain sensor is easy to produce, using a facile and solvent-free contact printing method; (3) Due to the unique microphase separation properties of the PU film, its rippled surface topology contributes to high sensitivity; (4) It bonds well with hydrogels, facilitating the integration of sensor components with the biomimetic 3D environment. Furthermore, the CS-HA membrane used in this study not only facilitates the assembly of 2D cardiomyocytes into 3D cardiomyocyte spheroids but also supports future developments toward 3D coculture systems (i.e., coculture of cardiomyocytes with other cell types). The integration of these advances is set to improve the development of next-generation biomedical devices, paving the way for more complex and accurately biomimetic systems in tissue engineering.

## 5 Materials and Methods

### 5.1 Synthesis and Characterization of the GNPs

The synthesis of GNPs was adapted from the method described by Peng et al.,^67^ with certain alterations. Initially, a precursor solution was prepared by mixing 100 mg of chloroauric acid trihydrate (99.99%, Alfa Aesar), 5.66 g of 1-dodecylamine (98%, Sigma-Aldrich), and 10 mL of hexane (≥99%, VWR Chemicals). The mixture was stirred magnetically at 300 rpm under a nitrogen atmosphere at a temperature of 30 °C. Subsequently, the stirring speed was increased to 1000 rpm, and a reduction solution containing 44 mg of *tert*-butylamine-borane complex (98%, Merck), 0.56 g of 1-dodecylamine, and 1 mL of hexane was quickly injected into this precursor mixture. After 1 h at a fixed stirring speed of 1000 rpm, 50 mL of ethanol (≥99.9%, VWR Chemicals) was added to induce the precipitation of the GNPs. The nanoparticles were then centrifuged at 20 000 g for 10 min. The supernatant was decanted, and the precipitate was dried under nitrogen flow for 1 h. Next, 4 mL of *n*-heptane (>99%, Honeywell) was added to dissolve the nanoparticles, followed by the addition of 12 mL of 2-propanol (≥99.5%, VWR Chemicals). The mixture was centrifuged again at 20000 g for 10 min, and the supernatant was decanted. This step, involving the addition of *n*-heptane and 2-propanol followed by centrifugation, was performed twice. Finally, the supernatant was decanted, the precipitate was dried using nitrogen flow for 1 h, and the nanoparticles were dissolved in 4 mL of *n*-heptane to form the GNP stock solution (with a particle concentration of ∼9 μM).^68^ For characterization, the GNPs were analyzed by TEM, using a Jeol JEM-1011 microscope with an acceleration voltage of 100 kV. Before the TEM analysis, 1-dodecylamine was exchanged for 1-dodecanethiol to enhance the stability of the GNPs. To 50 μL of the GNP stock solution, 10 μL (42 μmol) of 1-dodecanethiol and 500 μL of *n*-heptane were added. After shaking at 400 rpm for 1 h, 1 mL of ethanol was added, and the obtained precipitate was isolated by centrifugation at 5500 rpm for 10 min. The precipitate was resuspended in 1 mL of ethanol and again isolated by centrifugation. Subsequently, the precipitate was dried under nitrogen flow for 1 h and redissolved in 2 mL of *n*-heptane. This solution was used to transfer the GNPs onto a carbon-coated TEM copper grid for TEM analysis. The average diameter of the GNPs was determined using ImageJ software.

### 5.2 Fabrication and characterization of the GNP-PU film

The fabrication of the GNP film was conducted using the layer-by-layer spin-coating technique as described previously.^3,19^ Initially, a glass coverslip was thoroughly cleaned by sonicating (Bandelin Sonorex RK 255 H) it in acetone for 15 min, followed by rinsing with deionized water, and dried under ambient air. The glass was then subjected to air plasma treatment for 12 min using a plasma cleaner (PDC-32G, Harrick Plasma) at 18W. After waiting for 1 day, the glass slide was positioned at the center of a spin-coater and spun at a speed of 3000 rpm. Fifteen μL of 9DT (97%, Alfa Aesar) was dissolved in 10 mL of methanol (≥99.9%, VWR Chemicals) to obtain a concentration of 7.4 mM for the 9DT linker solution. Then, 100 μL of the 9DT linker solution was dropped twice onto the spinning glass slide. Subsequently, 10 μL of GNP solution and 2 × 10 μL of 9DT linker solution were alternatingly dropped onto the rotating glass slide. Each deposition step was spaced by ∼30 s. This cycle was repeated three times to achieve the desired film thickness. Finally, the glass slide covered with the GNP film was immersed overnight in the 9DT linker solution, then cleaned with acetone, and left to dry in ambient air for a day.

The biocompatible PU film was prepared as reported by Brinkman et al. with some modifications.^69^ Pellethane 2363 80A (Lubrizol) was first dissolved in tetrahydrofuran (≥99.7%, VWR Chemicals) to create a 4.5% (w/w) solution. This solution was shaken at room temperature for 6 h to ensure complete dissolution. Subsequently, the solution was passed through a 0.45 μm Teflon filter. The filtered solution was then cast onto the glass Petri dish. After the solvent evaporated for 1 day at 40 °C, the resulting film underwent further drying in a vacuum oven for 12 h at 60 °C. The dried film was then immersed in methanol for 48 h, with the methanol being replaced every 6 h. Finally, the thoroughly washed film underwent additional drying in a vacuum oven for another 12 h at 60 °C. The thickness of the prepared PU film was ∼230 μm.

The GNP-PU film was fabricated using a facile, manually performed contact printing method, as described in our previously published literature.^3,13^ Briefly, a PDMS elastomer was first fabricated by mixing a base polymer with a curing agent in a 10:1 weight ratio, using the Dow Sylgard 184 Kit Silicone Elastomer (Sigma-Aldrich). After curing the mixture for 4 h at 80 °C, a PDMS elastomer with a thickness of ∼5 mm was obtained. The elastomer was then cut with a sharp blade to form a PDMS stamp of the desired size. The role of the PDMS stamp was to serve as an intermediary step, allowing the GNP film to be transferred from glass to the PU film. To facilitate the transfer of the GNP film onto the final PU substrate, a heating and cooling cycle similar to the method described by Choi et al.^70^ was employed. Initially, the sample was placed on a hot plate (Präzitherm 2860SR), covered with a glass Petri dish (80 mm diameter), and maintained at 60 °C for 3 min. After that, the sample underwent a cooling process where it was brought down to 0 °C for another 3 min.

The surface morphology of the GNP films was analyzed before (i.e., on the glass) and after contact printing (i.e., on the PU film). This analysis was conducted using optical microscopy (Olympus BX51), SEM (Zeiss LEO Gemini 1550, at a 5 kV operating voltage in high vacuum mode), and AFM (Digital Instruments Multimode machine, which included an AppNano ACTA cantilever, a Veeco 100 μm scanner, and a Nanoscope IV controller). For AFM measurements, all scans were conducted in tapping mode and analyzed using the Gwyddion software.

### 5.3 Fabrication and Characterization of the GNP-PU Strain Sensor

The fabrication of the cantilever-based GNP-PU strain sensor involved a sequential process, as shown in Figure 3A. To obtain the GNP-PU strain sensor, gold electrodes were deposited onto the GNP-PU film using the PVD technique (Pfeiffer Classic 250). The PU (i.e., Pellethane 2363 80A) solution was used as the glue to adhere another PU film onto the GNP-PU strain sensor. To build the cantilever-based GNP-PU strain sensor, the Lab-Tek tissue culture chamber slide (Nunc) was employed and cut to create a slit (width and height of 10.5 mm and 0.6 mm, respectively). The epoxy glue (Wiko Epoxy 05) was used to fix the GNP-PU strain sensor onto the chamber slide. The length and width of the sensor inside the chamber were 12 mm and 10 mm, respectively.

The I–V curve of the GNP-PU strain sensor was measured by employing a Keithley 2601A sourcemeter using a voltage range from −5 to 5 V. Resistive strain responses were characterized by the bending tests and cyclic strain-relaxation test. The bending tests were performed with a custom-built four-point bending setup, as previously described.^37^ To test the impact of cell culture environments on the GNP-PU strain sensor, the sensor was fully bonded with the PU glue to another PU film and then placed in the cell culture medium (STEMdiff cardiomyocyte maintenance medium) for 2 days at 37 °C. The GNP-PU strain sensors, before and after medium treatment, were fixed onto the circuit board stripe (FR4, thickness of ∼0.8 mm) using 3 M Scotch adhesive (4004L 12). For comparison, a commercial strain gauge (SGT-1/350-TY11) with a gauge factor of 2.14 served as the reference sensor. The cyclic strain-relaxation test of the cantilever-based GNP-PU strain sensor was conducted in the cell culture medium using the custom-built bending setup, as shown in Figure 3Di. To establish electrical connections to sensors in both the bending and cyclic strain-relaxation tests, copper wires equipped with small crocodile clips were used. These wires were then connected to a sourcemeter (Keithley 2601A). The resistances of the sensors were measured by applying a constant voltage of 1.0 V and recording the resultant current. Both tests were conducted under ambient environmental conditions.

### 5.4 Preparation and Characterization of the Self-Healing Hydrogel

The self-healing hydrogel composed of CS-Ph and DF-PEG was prepared by following previous literature with modification.^47,48^ To synthesize CS-Ph, 200 mg of chitosan (molecular weight ∼145 kDa, degree of deacetylation ∼80%, Sigma) was initially dissolved in 2.0 mL of 1N hydrochloric acid (Showa) and then diluted with 8.0 mL of 50 mM 2-(N-morpholino)ethanesulfonic acid (MES) buffer solution (Sigma). At the same time, a combination of 99.7 mg of 3-(4-hydroxyphenyl)propionic acid (Alfa Aesar), 115.0 mg of 1-ethyl-3-(3-(dimethylamino)propyl)carbodiimide (Alfa Aesar), and 69.2 mg of *N*-hydroxysuccinimide (Sigma) was sequentially added to 90 mL of MES buffer. The two separately prepared solutions were then uniformly mixed and the pH was adjusted to 4. The reaction was carried out in a dark environment at a temperature of 25 °C for 24 h with constant stirring. Postreaction, the mixture was dialyzed using a dialysis membrane with a 12–14 kDa cutoff in deionized water at least five times to remove the unreacted chemicals. Finally, the purified CS-Ph product was obtained after freeze-drying.

For the DF-PEG synthesis, 2 g of PEG (molecular weight ∼8 kDa, Sigma) was initially dissolved in 100 mL of anhydrous tetrahydrofuran (Echo). Sequentially, 375.0 mg of 4-formylbenzoic acid (Sigma), 170.0 mg of 4-(dimethylamino)pyridine (Sigma), and 1.0 g of N,*N*′-dicyclohexylcarbodiimide (Sigma) were added to the PEG solution. The mixture was reacted at 25 °C for 48 h. Subsequently, the reacted solution was added to 300 mL of diethyl ether (Echo). The resulting white precipitate was filtered and then dissolved in 100 mL of tetrahydrofuran. The product, DF-PEG, was obtained as a white solid after it underwent three repeated cycles of precipitation in diethyl ether and redissolution in tetrahydrofuran. The synthesized CS-Ph and DF-PEG were both dissolved in the cell culture medium (STEMdiff cardiomyocyte maintenance medium). The self-healing hydrogel was produced by mixing the CS-Ph solution (2 wt %) with DF-PEG solution (4 wt %) at the volume ratio of 1:1.

### 5.5 Preparation of 3D Cardiomyocyte Spheroids

The guideline protocol of the STEMdiff cardiomyocyte differentiation kit was followed to produce a 2D hiPSC-derived cardiomyocyte sheet. Briefly, hiPSCs were initially cultured on a Matrigel-coated dish with mTeSR1 medium to reach ∼95% confluency. The cardiac differentiation began (day 0) by treating the cells with cardiomyocyte differentiation medium A for 2 days, followed by medium B for 2 days, and finally medium C for another 4 days. After differentiation, the cells were maintained in STEMdiff cardiomyocyte maintenance medium. The culture environment was a humidified incubator at 37 °C with 5% CO_2_, and the medium was refreshed daily. On day 19, the beating 2D hiPSC-derived cardiomyocyte sheet was obtained.

The CS-HA composite membrane was prepared as previously reported,^52^ prior to the production of 3D cardiomyocyte spheroids. Briefly, the chitosan (510 kDa, degree of deacetylation ∼77%, Sigma) solution was first coated onto the 24-well tissue culture plates. After evaporating the solvent in a laminar flow cabinet overnight, the chitosan membranes were soaked in 0.5 N sodium hydroxide for 30 min and washed thrice with phosphate buffered saline (PBS). Hyaluronan (1800 kDa, SciVision Biotech) solution was then added to the chitosan-coated plates and evaporated in a laminar flow cabinet overnight. Subsequently, the membranes were cross-linked with a 1-ethyl-3-(3-(dimethylamino)propyl)carbodiimide/*N*-hydroxysuccinimide solution for 48 h. After cross-linking, the fabricated CS-HA membranes were washed thoroughly with PBS thrice to remove any unbound hyaluronan and stored at 4 °C before use.

The 3D cardiomyocyte spheroids were fabricated using the 2D hiPSC-derived cardiomyocyte sheet and the fabricated CS-HA membrane. Initially, the cardiomyocyte sheet was rinsed with Dulbecco’s phosphate-buffered saline and then dissociated using the STEMdiff dissociation kit. Subsequently, the dissociated single-cell cardiomyocytes were seeded onto the CS-HA membrane with a density of 3.8 × 10^5^ cells per well. After culturing for 3 days, the 3D cardiomyocyte spheroids were obtained.

### 5.6 Flexible Electronic System

The flexible electronic system integrated the cell-laden gel matrix with the cantilever-based GNP-PU strain sensor. To build the cell-laden gel matrix, 3D cardiomyocyte spheroids were embedded into the self-healing hydrogel with a density of 1 × 10^7^ cells per mL. Subsequently, the cell-laden gel matrix was cultured on the cantilever-based GNP-PU strain sensor for 28 days. The copper wires equipped with small crocodile clips were sterilized and used to connect the sensor and the sourcemeter (Keithley 2601A) for detecting the contractile behavior of cardiomyocyte spheroids. The viability of cardiomyocyte spheroids was evaluated using LIVE/DEAD Cell Vitality Kits (Invitrogen, USA). The spheroid-laden gel matrix was washed with Dulbecco’s PBS (DPBS) and then immersed in a solution of calcein AM and ethidium homodimer-1 for 15 min. Samples were excited at wavelengths of 488 and 514 nm and visualized using a Nikon Eclipse 80i fluorescent microscope (Tokyo, Japan). For immunostaining, cardiomyocyte spheroids were fixed with 4% paraformaldehyde (Sigma-Aldrich) for 15 min, permeabilized with 0.1% Triton X-100 (Sigma-Aldrich) in DPBS for 20 min, washed with DPBS, and blocked with 2% bovine serum albumin (Sigma-Aldrich) for 1 h. The antibody cardiac troponin T (Sigma-Aldrich) was added and incubated overnight at 4 °C. Afterward, the samples were washed three times with 1% TWEEN 20 (Sigma-Aldrich) in DPBS for 1 h, followed by the addition of the antimouse Alexa Fluor 488 secondary antibody and incubation for 1 h. The 3D distribution of the cardiomyocyte spheroids within the self-healing hydrogel was then visualized using confocal laser scanning microscopy (Leica TCS SP5 II). Besides, the cantilever-based control sensor replacing the GNP film with a nonstrain-responsive surface mount resistor (∼5.1 MΩ) was fabricated. The carbon conductive adhesive (EM-Tec) and epoxy glue (Wiko Epoxy 05) were used to fix the resistor on the control sensor.
