# Supplementary material for: Motion Sensing by a Highly Sensitive Nanogold Strain Sensor in a Biomimetic 3D Environment
Source: ACS Appl Mater Interfaces. 2024 Sep 10;16(42):56599–610. doi: 10.1021/acsami.4c08105 (PMC11503636; doi:10.1021/acsami.4c08105)
Supplement: Supplementary file 1 — am4c08105_si_001.pdf [file am4c08105_si_001.pdf]

Supporting Information

Motion Sensing by a Highly Sensitive Nanogold Strain Sensor in a Biomimetic 3D Environment

Shin-Da Wu[1,2], Horst Weller[2,4], Tobias Vossmeyer[2,*], and Shan-hui Hsu[1,3,*]

[1] Institute of Polymer Science and Engineering, National Taiwan University, No. 1, Sec. 4 Roosevelt Road, Taipei 10617, Taiwan

[2] Institute of Physical Chemistry, University of Hamburg, Grindelallee 117, Hamburg 20146, Germany

[3] Institute of Cellular and System Medicine, National Health Research Institutes, Miaoli 350401, Taiwan

[4] Fraunhofer Center for Applied Nanotechnology CAN, Grindelallee 117, Hamburg 20146, Germany

Pages: 6

Supplemental Figures: 5 (Figure S1-Figure S5)

Supplemental Table: 1 (Table S1)

*** Corresponding author:**

Shan-hui Hsu (ORCID: 0000-0003-3399-055X)

E-mail: shhsu@ntu.edu.tw

Tobias Vossmeyer (ORCID: 0000-0001-9738-3826)

E-mail: tobias.vossmeyer@chemie.uni-hamburg.de

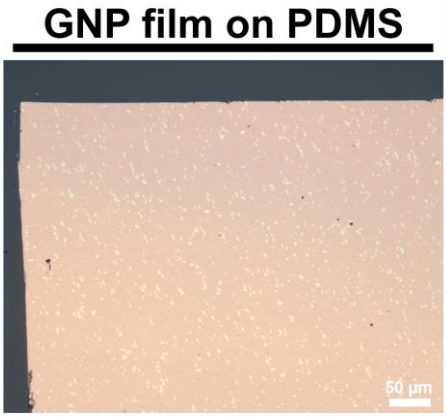

Figure S1. The GNP film transferred to the PDMS stamp showed its surface morphology in the OM image.

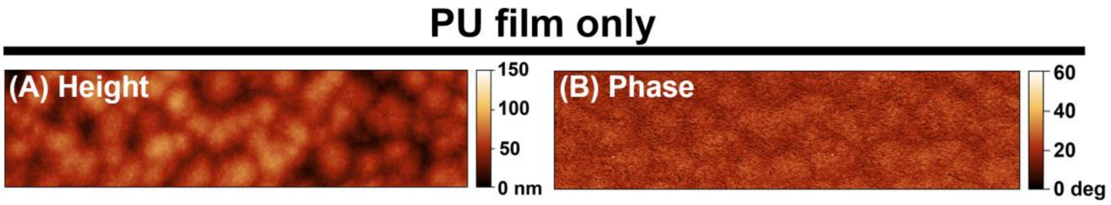

Figure S2. AFM images of the PU film. (A) Topographical image. (B) Phase image.

3D cardiac tissues			Measurements	Contractile stress	References
Heart muscles			Optical method	40-80 kPa	[59]
Cardiomyocytes	+	Fibrin hydrogel	Optical method	2-4 kPa	[56]
Cardiomyocytes	+	Collagen/Matrigel	Bioreactor force sensor	~5 kPa	[57]
Cardiomyocytes	+	Fibrinogen/Matrigel	Optical method	~28 kPa	[58]
Cardiomyocyte spheroids	+	Chitosan-based hydrogel	GNP-PU strain sensor	~20 kPa	This work

Table S1. Comparison of the contractile force in the present study with that for different types of 3D cardiac tissues reported in the published literature.

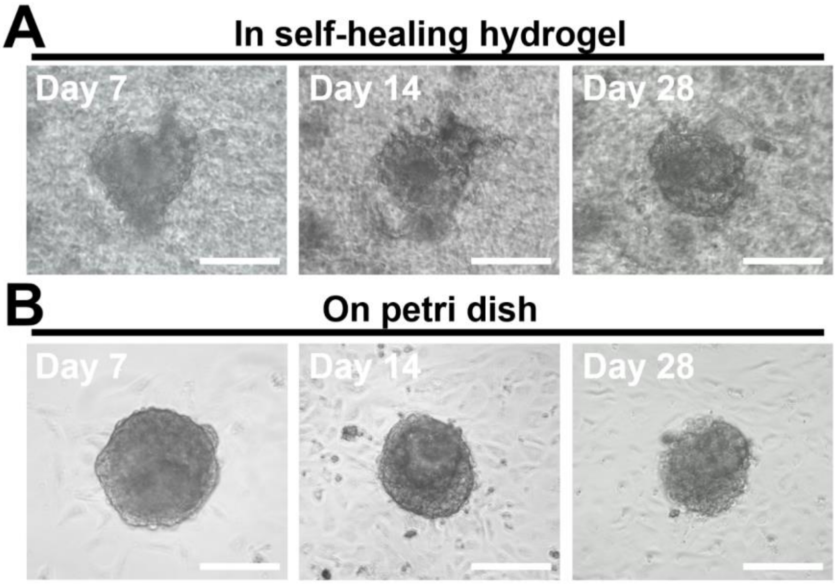

Figure S3. Changes in morphology of cardiomyocyte spheroids (A) in the self-healing chitosan-based hydrogel and (B) on the petri dish at different time points. Scale bars represent 100 μm.

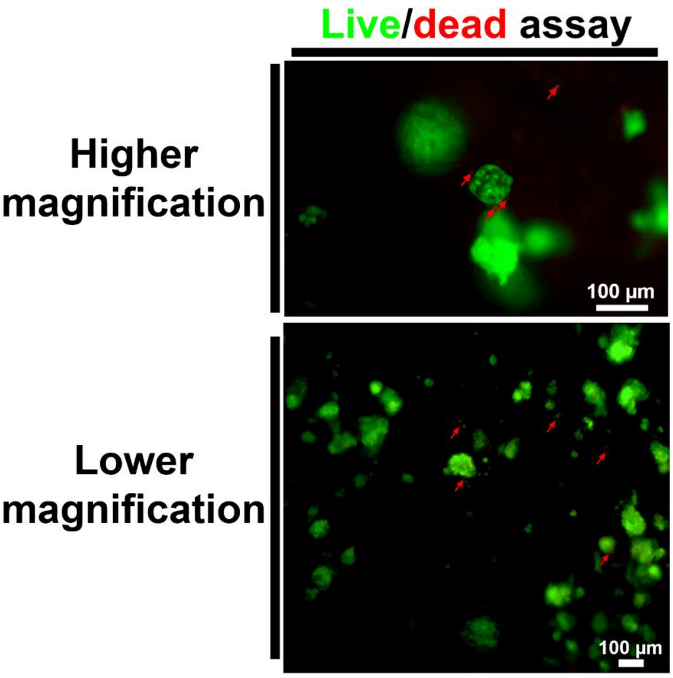

Figure S4. High and low magnification fluorescent microscopy images showing the live/dead staining of cardiomyocyte spheroids embedded within the self-healing hydrogel after 28 days of culture. Viable cells are stained with calcein AM (green), while dead cells are stained with ethidium homodimer-1 (red), indicated by red arrows.

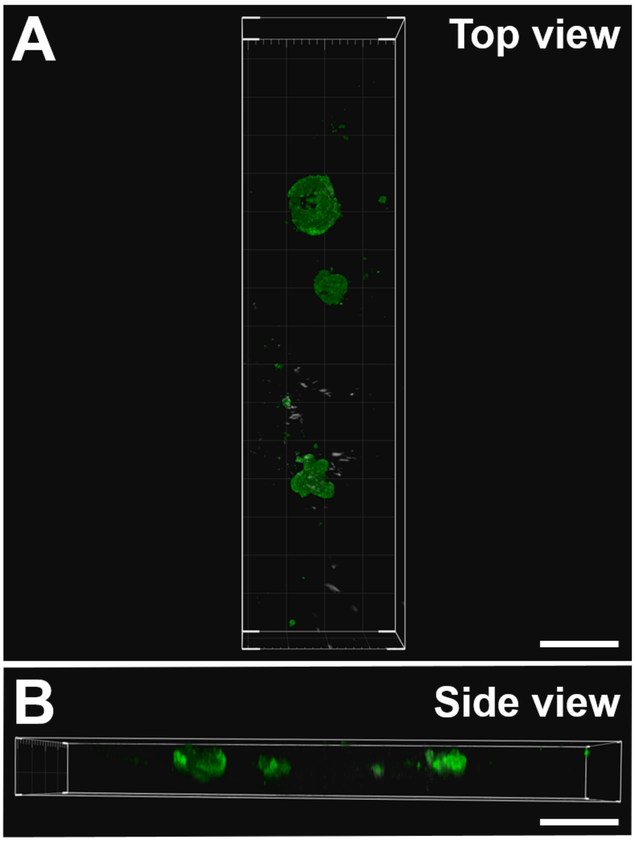

Figure S5. 3D confocal images of cardiomyocyte spheroids immunostained for cardiac troponin T within the self-healing hydrogel after 28 days of culture. (A) Top view. (B) Side view. Scale bars represent 100 μm.
